# Toward Computational Cumulative Biology by Combining Models of Biological Datasets

**DOI:** 10.1371/journal.pone.0113053

**Published:** 2014-11-26

**Authors:** Ali Faisal, Jaakko Peltonen, Elisabeth Georgii, Johan Rung, Samuel Kaski

**Affiliations:** 1 Helsinki Institute for Information Technology HIIT, Department of Information and Computer Science, Aalto University, Espoo, Finland; 2 European Molecular Biology Laboratory, European Bioinformatics Institute (EMBL-EBI), Wellcome Trust Genome Campus, Hinxton, United Kingdom; 3 Helsinki Institute for Information Technology HIIT, Department of Computer Science, University of Helsinki, Helsinki, Finland; University of South Florida, United States of America

## Abstract

A main challenge of data-driven sciences is how to make maximal use of the progressively expanding databases of experimental datasets in order to keep research cumulative. We introduce the idea of a modeling-based dataset retrieval engine designed for relating a researcher's experimental dataset to earlier work in the field. The search is (i) data-driven to enable new findings, going beyond the state of the art of keyword searches in annotations, (ii) modeling-driven, to include both biological knowledge and insights learned from data, and (iii) scalable, as it is accomplished without building one unified grand model of all data. Assuming each dataset has been modeled beforehand, by the researchers or automatically by database managers, we apply a rapidly computable and optimizable combination model to decompose a new dataset into contributions from earlier relevant models. By using the data-driven decomposition, we identify a network of interrelated datasets from a large annotated human gene expression atlas. While tissue type and disease were major driving forces for determining relevant datasets, the found relationships were richer, and the model-based search was more accurate than the keyword search; moreover, it recovered biologically meaningful relationships that are not straightforwardly visible from annotations—for instance, between cells in different developmental stages such as thymocytes and T-cells. Data-driven links and citations matched to a large extent; the data-driven links even uncovered corrections to the publication data, as two of the most linked datasets were not highly cited and turned out to have wrong publication entries in the database.

## Introduction

Molecular biology, historically driven by the pursuit of experimentally characterizing each component of the living cell, has been transformed into a data-driven science [1]–[6] with just as much importance given to the computational and statistical analysis as to experimental design and assay technology. This has brought to the fore new computational challenges, such as the processing of massive new sequencing data, and new statistical challenges arising from the problem of having relatively few (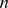) samples characterized for relatively many (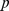) variables—the “large 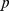, small 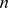
” problem. High-throughput technologies often are developed to assay many parallel variables for a single sample in a run, rather than many parallel samples for a single variable, whereas the statistical power to infer properties of biological conditions increases with larger sample sizes. For cost reasons, most labs are restricted to generating datasets with the statistical power to detect only the strongest effects. In combination with the penalties of multiple hypothesis testing, the limitations of “large 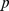, small 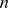
” datasets are obvious. It is, therefore, not surprising that much work has been devoted to address this problem.

Some of the most successful methods rely on increasing the effective number of samples by combining with data from other, similarly designed, experiments, in a large meta-analysis [7]. Unfortunately, this is not straightforward, either. Although public data repositories, such as the ones at NCBI in the United States and the EBI in Europe, serve the research community with ever-growing amounts of experimental data, they largely rely on annotation and meta-data provided by the submitter. Database curators and semantic tools such as ontologies provide some help in harmonizing and standardizing the annotation, but the user who wants to find datasets that are combinable with her own most often must resort to searches in free text or in controlled vocabularies, which would need significant downstream curation and data analysis before any meta-analysis can be done [8].

Ideally, we would like to let the data speak for themselves. Instead of searching for datasets that have been described similarly, which may not correspond to a statistical similarity in the datasets themselves, we would like to conduct that search in a data-driven way, using as the query the dataset itself or a statistical (rather than a semantic) description of it. This is implicitly done, for example, in multi-task learning, a method from the machine learning field [9], [10], where several related estimation tasks are pursued together, assuming shared properties across tasks. Multi-task learning is a form of global analysis, which builds a single unified model of the datasets. But as the number of datasets keeps increasing and the amount of quantitative biological knowledge keeps accumulating, the complexity of building an accurate unified model becomes increasingly prohibitive.

Addressing the “large 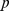, small 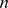
” problem requires taking into account both the uncertainty in the data and the existing biological knowledge. We now consider the hypothesized scenario where future researchers increasingly develop hypotheses in terms of (probabilistic) models of their data. Although far from realistic today, a similar trend exists for sequence motif data, which are often published as Hidden Markov models, for instance in the Pfam database [11].

In this paper, we report on a feasibility study that uses the scenario in which many experiments have been modeled beforehand, potentially by the researcher generating the data or automatically by the database storing the model together with the data. We ask *what could be done with these models towards cumulatively building knowledge from data in molecular biology*? Speaking about models generally and assuming the many practical issues can be solved technically, we arrive at our answer: we propose creating *a modeling-driven dataset retrieval engine*, which a researcher can use for positioning her own measurement data into the context of the earlier biology. The engine will point out relationships between experiments in the form of the retrieval results, which is a naturally understandable interface. The retrieval will be based on data, instead of the state-of-the-art practice of using keywords and ontologies, which will make unexpected and previously unknown findings possible. The retrieval will use the models of the datasets, which, by our assumption above, incorporate the knowledge of the researchers producing the data about what is important in the data, but the retrieval will be designed to be more scalable than building one unified grand model of all data. This also implies that the way the models are utilized needs to be approximate. Compared to existing data-driven retrieval methods [3], [5], whole datasets, incorporating the experimental designs, will be matched, instead of individual observations. The remaining question is how to design the retrieval so that it both reveals the interesting and important relationships and is fast to compute.

The model we present is a first step towards this goal. We assume that a new dataset can be explained by a combination of the models for the earlier datasets and a novelty term. This is a mixture modeling or regression task, in which the weights can be computed rapidly; the resulting method scales well to large numbers of datasets, and the speed of the mixture modeling does not depend on the sizes of the earlier datasets. The largest weights in the mixture model point at the most relevant earlier datasets. The method is applicable to several types of measurement datasets, assuming that suitable models exist. Unlike traditional mixture modeling, we do not limit the form of the mixture components; thus, we bring in the knowledge built into the stored models of each dataset. We apply this approach to a large set of experiments from EBI's ArrayExpress gene expression database [12], treating each experiment in turn as a new dataset, queried against all earlier datasets. Under our assumptions, the retrieval results can be interpreted as studies that the authors of the study generating the query set could have cited, and we show that the actual citations overlap with the retrieval results. The discovered links between datasets additionally enable forming a “hall of fame” of gene expression studies, containing the studies that would have been influential, assuming the retrieval system existed. The links in the “hall of fame” verify and complement the citation links: in our study, they revealed corrections to the citation data, as two frequently retrieved studies were not highly cited and turned out to have erroneous publication entries in the database. We provide an online resource for exploring and searching this “hall of fame”: http://research.ics.aalto.fi/mi/setretrieval.

Earlier work on relating datasets has provided partial solutions along this line, with the major limitation of being restricted to pairwise dataset comparisons, in contrast to the proposed approach of decomposing a dataset into contributions from a set of earlier datasets. Russ and Futschik [13] represented each dataset by pairwise correlations of genes, and used them to compute dataset similarities. This dataset representation is ill suited for typical functional genomics experiments, as a large number of samples is required to sensibly estimate gene correlation matrices. In addition, it makes the dataset comparison computationally expensive, as the representation is bulkier than the original dataset. In other works, specific case-control designs [14] or known biological processes [15] are assumed; we generalize by using decompositions over arbitrary models.

In summary, our work is the first approach that allows data-driven retrieval of relevant datasets by decomposing a query dataset into contributions from several earlier datasets, without requiring specific designs for the earlier datasets or their models. Unlike existing state-of-the-art retrieval, our approach is not limited to available dataset annotation. Unlike the Pfam database [11], we not only store models but use them in retrieval. Unlike existing data-driven approaches [3], [5] that match individual observations, we match whole datasets incorporating their experimental designs. We fully decompose datasets instead of only computing pairwise similarities, as in [13], and we allow decomposition over arbitrary models available for the datasets instead of requiring restricted settings, such as specific case-control designs [14] or known biological processes [15]. Unlike a hypothetical approach where a unified model of all data is built, our approach is fast and scalable to large data.

### Combination of Stored Models for Dataset Retrieval

Our goal is to infer data-driven relationships between a new “query” dataset 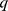 and earlier datasets. The query is a dataset of 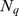 samples 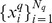; in the ArrayExpress study, the samples are gene expression profiles, with the element 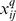 being expression of the gene set 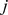 in the sample 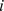 of the query 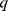, but the setup is general and applicable to other experimental data, as well. Assume further a dataset repository of 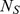 earlier datasets, and assume that each dataset 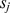, 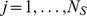, has already been modeled with a model denoted by 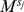, later called a base model. The base models are assumed to be probabilistic generative models, *i.e.*, principled data descriptions capturing prior knowledge and data-driven discoveries under specific distributional assumptions. Base models for different datasets may come from different model families, as chosen by the researchers who authored each dataset. In this paper, we use two types of base models, which are discrete variants of principal component analysis (*Results*), but any probabilistic generative models can be applied.

As an illustrative setting, suppose that the dataset repository contains several datasets arising from base experiments, so that each base experiment studies one known important biological effect, the experiment has been designed so that the effect is present in the resulting dataset, and together the base experiments cover the set of known important biological effects. In the special example case of metagenomics with known constituent organisms, an obvious set of base experiments would be the set of genomes of those organisms [16]. A new experiment could then be expressed as a combination of the base experiments, and potential novel effects. More generally, such as in a broad gene expression atlas, it would be hard, if not impossible, to settle on a clean, well-defined, and up-to-date base set of experiments to correspond to each known effect, so we chose to *use the comprehensive collection of experiments in the current databases as the base experiments*. The problem setting then changes from searching for a unique explanation of the new experiment to the down-to-earth and realistic task of data-driven retrieval of a set of relevant earlier experiments, relevant in the sense of having induced one or more of the known or as-of-yet unknown biological effects.

We combined the earlier datasets by a method that is probabilistic but simple and fast. We built a *combination model* for the query dataset as a mixture model of base distributions 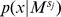, which have been estimated beforehand. In our scenario, generative models 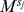 are available in the repository along with datasets 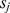; note that the 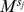 need not all have the same form. In the mixture model parameterized by 
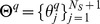
, the likelihood of observing the query is 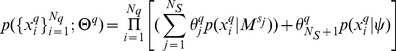
(1)where 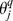 is the mixture proportion or *weight* of the 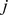
th base distribution (model of dataset 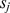), and 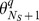 is the weight for the novelty term. The novelty is modeled by a background model 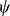, a broad nonspecific distribution covering overall gene-set activity across the whole dataset repository. All weights are non-negative and 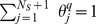. In essence, this representation assumes that biological activity in the query dataset can be approximately explained as a combination of earlier datasets and a novelty term.

The remaining task is to infer the combination model 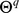 for each query 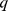 given the known models 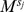 of datasets in the repository. We infer a maximum a posteriori (MAP) estimate of the weights 
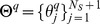
. Alternatively, we could sample over the posterior, but MAP inference already yielded good results. We optimize the combination weights to maximize their (log) posterior probability 
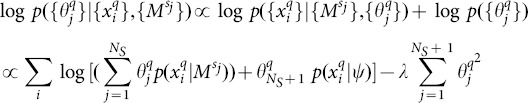
(2)where 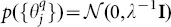 is a naturally non-sparse 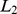 prior distribution for the weights with a regularization term 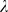. The cost function (2) is strictly concave (Text S1), and standard constrained convex optimization techniques can be used to find the optimized weights. Algorithmic details for the Frank-Wolfe algorithm and a proof of convergence are provided in Text S1. After computing the MAP estimate, we rank the datasets for retrieval according to decreasing combination weights.

This modeling-driven approach has several advantages: 1) the approximations become more accurate as more datasets are submitted to the repository, naturally increasing the number of base distributions; 2) it is fast, as only the models of the datasets are needed, not the large datasets themselves; 3) any model types can be included, as long as likelihoods of an observed sample can be computed; hence, all expert knowledge built into the models in the repository can be used; 4) relevant datasets are not assumed to be similar to the query in any na?ve sense, as they only need to explain a part of the query set; 5) the relevance scores of datasets have a natural quantitative meaning as weights in the probabilistic combination model.

### Scalability

As the size of repositories such as ArrayExpress doubles every two years or even more rapidly [17], fast computation with respect to the number 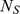 of background datasets is crucial for future-proof search methods. The first method above already has a fast linear computation time in 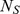 (Text S1), and an approximate variant can be run in sublinear time. For that, the model combination will be optimized only over the 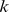 background datasets most similar to the query, which can be found in time 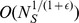 where 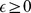 is an approximation parameter [18], by suitable hashing functions.

## Results

### Data-driven retrieval of experiments is more accurate than standard keyword search

**Figure 1:**
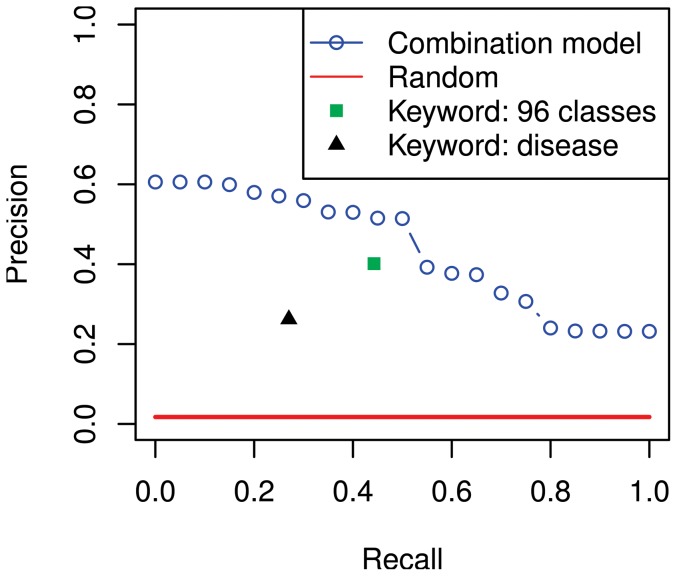
Data-driven retrieval outperforms the state of the art of keyword search on the human gene expression atlas [12]. Blue: Traditional precision-recall curve where progressively more datasets are retrieved from left to right. All experiments sharing one or more of the 96 biological categories of the atlas were considered relevant. In keyword retrieval, either the category names (“Keyword: 96 classes”) or the disease annotations (“Keyword: disease”) were used as keywords. All datasets having at least 10 samples were used as query datasets, and the curves are averages over all queries.

We benchmarked the combination model against state-of-the-art dataset retrieval by keyword search, in the scenario in which a user queries with a new dataset against a database of earlier released datasets represented by models. The data were from a large human gene expression atlas [12], containing 206 public datasets with 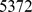 samples that have been systematically annotated and consistently normalized. To make use of prior biological knowledge, we preprocessed the data by gene set enrichment analysis [19], representing each sample by an integer vector telling for each gene set the number of leading edge active genes [20] (*Methods*). As base models, we used two model types previously applied in gene expression analysis [3], [6], [20], [21]: a discrete principal component analysis method called Latent Dirichlet Allocation [22], [23], and a simpler variant called mixture of unigrams [24] (Text S1). Of the two types, for each dataset, we chose the model yielding the larger predictive likelihood (Text S1). For each query (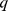), the earlier datasets (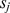) were ranked in descending order of the combination proportion (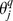; estimated from Eq. (2)). That is, base models that explained a larger proportion of the gene set activity in the query were ranked higher. The approach yields good retrieval: the retrieval result was consistently better than with keyword searches applied to the titles and textual descriptions of the datasets (Fig. 1), which is a standard approach for dataset retrieval from repositories [25].

We checked that the result was not only due to laboratory effects by discarding, in a follow-up study, all retrieved results coming from the same laboratory. The mean average precision decreased slightly (from 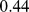 to 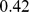; precision-recall curve in Fig. S2) but still supports the same conclusion.

### Network of computationally recommended dataset connections reveals biological relationships

**Figure 2:**
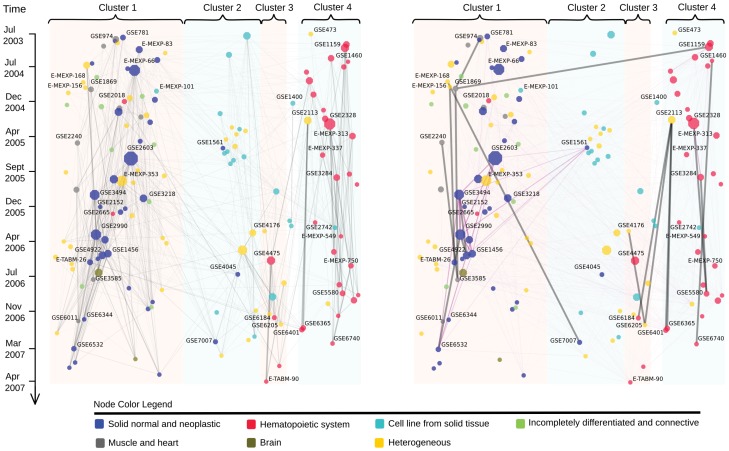
Relevance network of datasets in the human gene expression atlas; data-driven links from the model (left) and citation links (right). Left: each dataset was used as a query to retrieve earlier datasets; a link from an earlier dataset to a later one means the earlier dataset is relevant as a partial model of activity in the later dataset. Link width is proportional to the normalized relevance weight (combination weight 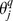; only links with 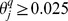 are shown, and datasets without links have been discarded). Right: links are direct (gray) and indirect (purple) citations. Node size is proportional to the estimated influence, *i.e.*, the total outgoing weight. Colors: tissue types (six meta tissue types [12]). The node layout was computed from the data-driven network (details in *Methods*).

When each dataset in turn is used as a query, the estimated combination weights form a “relevance network” between datasets (Fig. 2, left), where each dataset is linked to the relevant earlier datasets (for details, see *Methods* and an interactive searchable version at http://research.ics.aalto.fi/mi/setretrieval). The network structure is dominated but not fully explained by the tissue type. Normal and neoplastic solid tissues (cluster 1) are clearly separate from cell lines (cluster 2) and from hematopoietic tissue (cluster 4); the same main clusters were observed in [12]. Note that the model has not seen the tissue types but has found them from the data. Upon closer inspection of the clusters, some finer structure is evident. The muscle and heart datasets (gray) form an interconnected subnetwork in the left edge of the image: nodes near the bottom of the image (downstream) are explained by earlier (upstream) nodes, which in turn are explained by nodes even further upstream. As another example, in cluster 4, myeloma and leukemia datasets are concentrated on the left side of the cluster, whereas the right side mostly contains normal or infected mononuclear cells.

There is a substantial number of links both across clusters and across tissue categories. Among the top 30 cross-category links, 25 involve heterogeneous datasets containing samples from diverse tissue origins. The strongest link connects GSE6365, a study on multile myeloma, with GSE2113, a larger study from the same lab, which largely includes the GSE6365 samples. The dataset E-MEXP-66 is a hub connected to all of the clusters and to nodes in its own cluster that have different tissue labels. It contains samples studying Kaposi sarcoma, and it also includes control samples from skin endothelial cells from blood vessels and the lymph system. Blood vessels and cells belonging to the lymph system are expected to be present in almost any solid tissue biopsy as well as in samples based on blood samples. The strongest link between two homogeneous datasets of different tissue types connects GSE3307, which compares skeletal muscle samples from healthy individuals with 12 groups of patients affected by various muscle diseases, to GSE5392, which measures the transcriptome profiles of the normal brain and a brain with bipolar disorder. Interestingly, the shortening of telomeres has been associated both with bipolar disorder [26] and muscular disorder [27]. Treatment of bipolar disorder has been found to also slow down the onset of skeletal muscle disorder [28].

Next, we investigated “outlier" datasets where the tissue type does not match the main tissue types of a cluster, implying that they might reveal commonalities between cellular conditions across tissues. Cluster 1 contained three outlier datasets: two hematopoietic datasets and one cell line dataset. The two hematopoietic outlier datasets are studies related to macrophages and are both strongly connected to GSE2004, which contains samples from the kidney, liver, and spleen, sites of long-lived macrophages. The first hematopoietic outlier, GSE2018, studies bronchoalveolar lavage cells from lung transplant receipts; the majority of these cells are macrophages. The dataset has strong links to solid tissue datasets, including GSE2004, and the diverse dataset E-MEXP-66. The second hematopoietic outlier, GSE2665, is also strongly connected to GSE2004 and measures the expression of the lymphatic organs (sentinel lymph node) that contain sinusoidal macrophages and sinusoidal endothelial cells. The third outlier, E-MEXP-101, studies a colon carcinoma cell line and has connections to other cancer datasets in cluster 1.

### Top dataset links overlap well with citation graph

We compared the model-driven network to the actual citation links (Fig. 2, right) to find out to what extent the citation practice in the research community matches the data-driven relationships. Of the top 200 data-driven edges, 50% overlapped with direct or indirect citation links (see *Methods*, Text S1 and Fig. S3). Most of the direct citations appear within the four tissue clusters (Fig. 2, right). The two cross-cluster citations are not due to the biological similarity of the datasets. The publication for GSE1869 cites the publication for GSE1159 regarding the method of differential expression detection. The GSE7007, a study on Ewing sarcoma samples, cites the study on human mesenchymal stem cells (E-MEXP-168), stating that the overall gene expression profiles differ between those samples.

We additionally compared the densely connected sets of experiments between the two networks. In the citation graph, the breast cancer datasets GSE2603, GSE3494, GSE2990, GSE4922, and GSE1456 form an interconnected clique in cluster 1, while the three leukocyte datasets GSE2328, GSE3284, and GSE5580 form an interconnected module in cluster 4. In the relevance network, the corresponding edges for both cliques are among the strongest links for those datasets, and some of them are among the top 20 strongest edges in the network (see Table S1 for the list of top 20 edges). There are also densely connected modules in the relevance network that are not strongly connected in the citation graph; when we systematically sought cliques associated with each of the top 20 edges, the strongest edges constitute a clique among E-MEXP-750, GSE6740, and GSE473, all three studying CD4+ T helper cells, which are an essential part of the human immune system. Another interesting set is among three T-cell related datasets in cluster 3. Two of the datasets contain T lymphoblastic leukemia samples (E-MEXP-313 and E-MEXP-549), whereas E-MEXP-337 reports thymocyte profiles. Thymocytes are developing T lymphocytes that are matured in thymus, so this connection is biologically meaningful but not straightforward to find from dataset annotations. Other strongly connected cliques are discussed in Text S1.

### Analysis of network hubs discovers datasets deserving more citations

**Figure 3:**
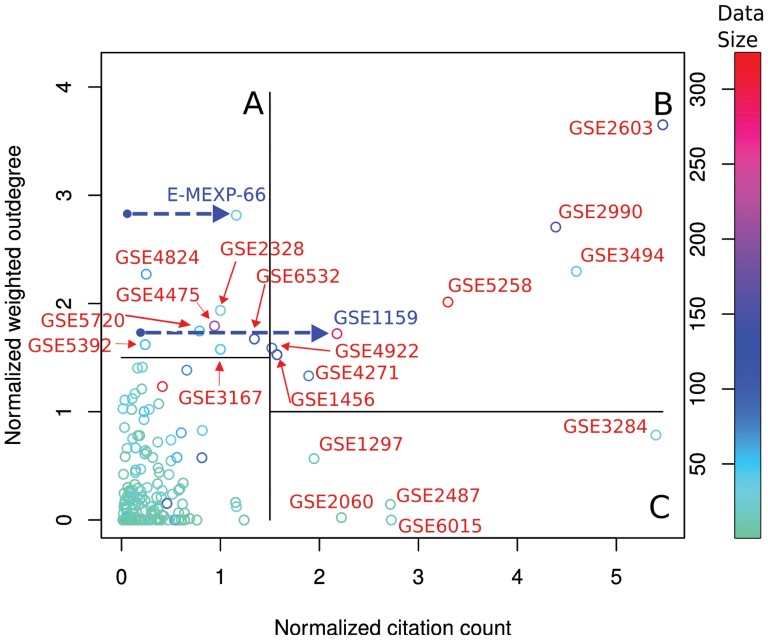
Data-driven prediction of usefulness of datasets vs. their citation counts. Manual checks comparing sets for which the two scores differed revealed inconsistent database records for two datasets; the blue arrows point to their corrected locations, which are more in line with the data-driven model. Regions A, B, and C: see text.

Datasets that have high weights in explaining other datasets have a large weighted outdegree in the data-driven relevance network, and they are expected to be useful for many other studies. We checked whether the publications corresponding to these *central hubs* are highly cited in the research community. There is a low but statistically significant correlation between the weighted outdegree of datasets and their citation counts (Fig. 3; Spearman 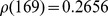, 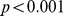). Both quantities were normalized to avoid bias due to different release times of the datasets (*Methods*). We further examined whether the prestige of the publication venue (measured by impact factor) and the senior author (h-index of the last author) biased the citation counts, which could explain the low correlation between the outdegree and the citation count, and the answer was affirmative (*Methods*).

We inspected more closely the datasets where the recommended or the actual citation counts were high (Fig. 3): (A) datasets having low citation counts but high outdegrees, (B) datasets having both high citation counts and high outdegrees, and (C) datasets having high citation counts but low outdegrees. We manually checked the publication records of region A in Gene Expression Omnibus (GEO) [29] and ArrayExpress [17], to find out why the datasets had low citation counts despite their high outdegree (data-driven citation recommendations). Two of the eight datasets had an inconsistent publication record. The blue arrows in Fig. 3 point from their original position to the corrected position confirmed by GEO and ArrayExpress. Thus, the data-driven network revealed the inconsistency, and the new positions, corresponding to higher citation counts, validate the model-based finding that these datasets are good explainers for other datasets. In region B, most of the papers have been published in high-impact journals and have a relatively high number of samples (average sample size of 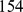) compared to region A (average sample size of 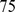). One of the eight datasets in the collection is the well-known Connectivity Map experiment (GSE5258). Lastly, the set C mostly contains unique targeted studies; there are five studies in the set, which are about leukocytes of injured patients, Polycomb group (PcG) proteins, senescence, Alzheimer's disease, and the effect of cAMP agonist forskolin, a traditional Indian medicine. The studies have been published in high-impact forums, and a possible reason of their low outdegree is their specific cellular responses, which are not very common in the atlas.

## Discussion

Our main goal was to test the feasibility of the scenario where researchers let the data speak for themselves when relating new research to earlier studies. The conclusion is positive: even a relatively straightforward and scalable mixture modeling approach found both expected relationships such as tissue types, and relationships not easily found with keyword searches, including cells in different developmental stages or treatments resembling conditions in other cell types. While biologists could find such connections by bringing expert knowledge into keyword searches, the ultimate advantage of the data-driven approach is that it also yields connections beyond current knowledge, giving rise to new hypotheses and follow-up studies. For example, it seems surprising that the skeletal muscle dataset GSE6011 is linked also to kidney and brain datasets. Closer inspection yielded possible partial explanations. Some kidney areas are rich in blood vessels, lined by smooth muscle. Studies have shown common gene signatures between skeletal muscle and brain. Abnormal expression of the protein dystrophin leads to Duchenne muscular dystrophy, exhibited by a majority of samples in GSE6011; the brain is another major expression site for dystrophin [30]. Interestingly, the top three potentially novel datasets, where only less than 50% of the expression pattern is modelled by earlier datasets (i.e., 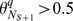), are GSE2603 (a central breast cancer set), the Connectivity Map data (GSE5258), and the Burkitt's Lymphoma set (GSE4475, a cancer fundamentally distinct from other types of lymphoma). The first two are also recovered by the citation data (as they have relatively high citation counts and appear in region B in Fig. 3), unlike the third (which is part of region A in Fig. 3).

Our case study focused on a global analysis of the relevance network obtained for a representative dataset collection, allowing for comparisons with the citation graph. The data-driven relationships corresponded to actual citations when available but were richer and were able to spot out errors in citation links. Another intended use of the retrieval method is to support researchers in finding relevant data on a particular topic of interest. We performed a study with additional skeletal muscle datasets (Table S2) to obtain insights into relationships among skeletal muscle datasets (Text S1) as well as between skeletal muscle and other datasets (Text S1 and Table S3), and we showed that the retrieval method lessens the need for laborious manual searches (Text S1 and Fig. S4).

In this work, we made simplifying assumptions: we only employed two model families, included biological knowledge only as pre-chosen gene sets, and assumed all new experiments to be mixtures of earlier ones, instead of finding common effects in them and combining them either as mixtures or sums. We expect the results to improve considerably with more advanced future alternatives, with the research challenge being to maintain scalability. Generalizability of the search across measurement batches, laboratories, and measurement platforms is a challenge. Our feasibility study showed that for carefully preprocessed datasets (of the microarray atlas [12]), data-driven retrieval is useful even across laboratories. Our method is generally applicable to any single platform, and it takes into account the expert knowledge built into models of datasets for that platform; abstraction-based data representations, such as the gene set enrichment representation we used, have the potential to facilitate cross-platform analyses. As data integration approaches develop further [31], [32], it may be possible to do searches even across different omics types; here, integration of meta data (pioneered in a specific semi-supervised framework [33]), several ontologies (MGED ontology, experimental factor ontology, and ontology of biomedical investigations [34]) and text mining results [35], [36] are obviously useful first steps.

## Materials and Methods

### Gene expression data

We used the human gene expression atlas [12] available at ArrayExpress under accession number E-MTAB-62. The data were preprocessed by gene set enrichment analysis (GSEA) using the canonical pathway collection (C2-CP) from the Molecular Signatures Database [19]. Each sample was represented by its top enriched gene sets [20] (Text S1).

### Node layout and normalized relevance weight

The weight matrix contains a weight vector for each query dataset, encoding the amount of variation in that query explained by each earlier dataset. As query datasets from early years have only a few even earlier sets available, there is a bias towards the edges being stronger for the datasets from early years. To remove the bias we normalized, for the visualizations, the edge strengths of each query data set by the number of earlier datasets. To visualize the relationship network over time in Fig. 2, we needed a layout algorithm that positions the datasets on the horizontal axis highlighting structure and avoiding tangling. We used a *cluster-emphasizing* Sammon's mapping; Sammon's mapping [37] is a nonlinear projection method or multidimensional scaling algorithm that aims at preserving the interpoint distances (here 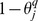). By clustering the network (with unsupervised Markov clustering [38]) and increasing between-cluster distances by adding a constant (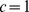) to them, the mapping was made to emphasize clusters and hence untangle the layout.

### Citation graph

Direct citations between dataset-linked publications were extracted from the Web of Science (26 Jul 2012) and PubMed (17 Oct 2012). We additionally considered two types of indirect edges. Firstly, we introduced links between datasets whose publications share common references. This covers, for instance, related datasets whose publications appeared close in time, making direct citation unlikely. A natural measure of edge strength is given by the number of shared references. Secondly, we connect datasets whose articles are cited together, because co-citation is a sign that the community perceives the articles as related. Here, the edge strength was taken to be the number of articles co-citing the two dataset publications; these edges dominate the indirect links in the citation graph. For this analysis, we used citation data, available for 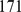 datasets and provided by Thomson Reuters as of 13 September 2012.

### Normalization of citation counts and weighted outdegrees

As early datasets have many more papers that can cite them and many more later datasets that they can help model, both the citation counts and estimated weighted outdegrees are expected to be upwards biased for them. For Fig. 3, we normalized the quantities; for each dataset, we normalized the outdegree by the number of newer datasets and the citation count by the time difference between publishing the data and the newest dataset in the atlas. To make sure the normalization did not introduce side effects, we additionally checked that the same conclusions were reached without the citation count normalization (Fig. S1; plotted as stratified subfigures for each 1-year time window). The citation counts were extracted from PubMed on 16 May 2012.

### Citation counts are strongly influenced by external esteem of the publication forum and the senior author

We stratified the data sets according to the numbers of data-driven citation recommendations, and studied whether the impact factor of the forum or the h-index of the last author were predictive of the actual citation count in each stratum. The strata were the top and bottom quartiles, and for each, we compared the top and bottom quartiles of the actual citation counts (resulting in comparing the four corners of Fig. 3). For low outdegree (low recommended citation count), the h-index was lower for less cited datasets (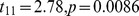; mean value 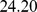 vs 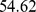), and the impact factor was lower (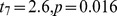; mean value 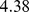 vs 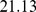). Similarly, for the high recommended citation count, the impact factor for the little-cited datasets was lower (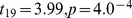; mean value 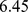 vs 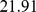), while the difference in h-index was not significant. All t statistics and p-values were computed by one-sided independent sample Welch's t-tests. The h-indices and impact factors were collected from Thomson Reuters Web of Knowledge and Journal Citation Reports 2011, respectively, on 23rd July 2012.

## Supporting Information

Figure S1 **Stratified data-driven prediction of usefulness of datasets vs. their citation counts.** Black solid lines mark the boundary for potentially interesting datasets; the boundaries are set to hold the same percentiles of data as in Fig. 3 in the main paper. *ImpFac* stands for Impact Factor of the publication venue.
(TIFF)

Figure S2 **Removal of laboratory effects changes the retrieval performance only slightly, as measured by the precision-recall curves.** *Original*: Replicated from Fig. 1 of the main paper; *Lab. effects removed*: all retrieval results from the same laboratory as the query data have been discarded.
(TIFF)

Figure S3
**Overlap of data-driven recommendations with the actual citation graph: Precision** 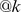 **for top edges that explain more than** 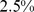 **variation.** The gold standard is the extended citation graph, which is built as the union of edges from 1) the original directed graph, 2) between any two articles that are cited together by some other article, and 3) between any two articles that have at least one common reference.(TIFF)

Figure S4 **Retrieval performance evaluation of the data-driven model against keyword search in the skeletal muscle case study.** The precision-recall curves are averaged across the 16 skeletal muscle datasets having at least 10 samples.
(TIFF)

Table S1
**Top** 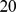 **strongest edges in the relevance network.**
(XLSX)

Table S2 **ArrayExpress accession numbers of 16 skeletal muscle datasets used in the retrieval case study in addition to the human gene expression atlas** [12] **.** All datasets were measured with the human genome platform HG-U133A, the same used in the atlas.
(XLSX)

Table S3 **Skeletal muscle queries with at least one retrieved non-skeletal muscle dataset, sorted according to decreasing precision.**
(XLSX)

Text S1 **More details on methods and results.**
(PDF)
