# Supplementary figures and images for: Toward Computational Cumulative Biology by Combining Models of Biological Datasets
Source: PLoS One. 2014 Nov 26;9(11):e113053. doi: 10.1371/journal.pone.0113053 (PMC4245117; doi:10.1371/journal.pone.0113053)

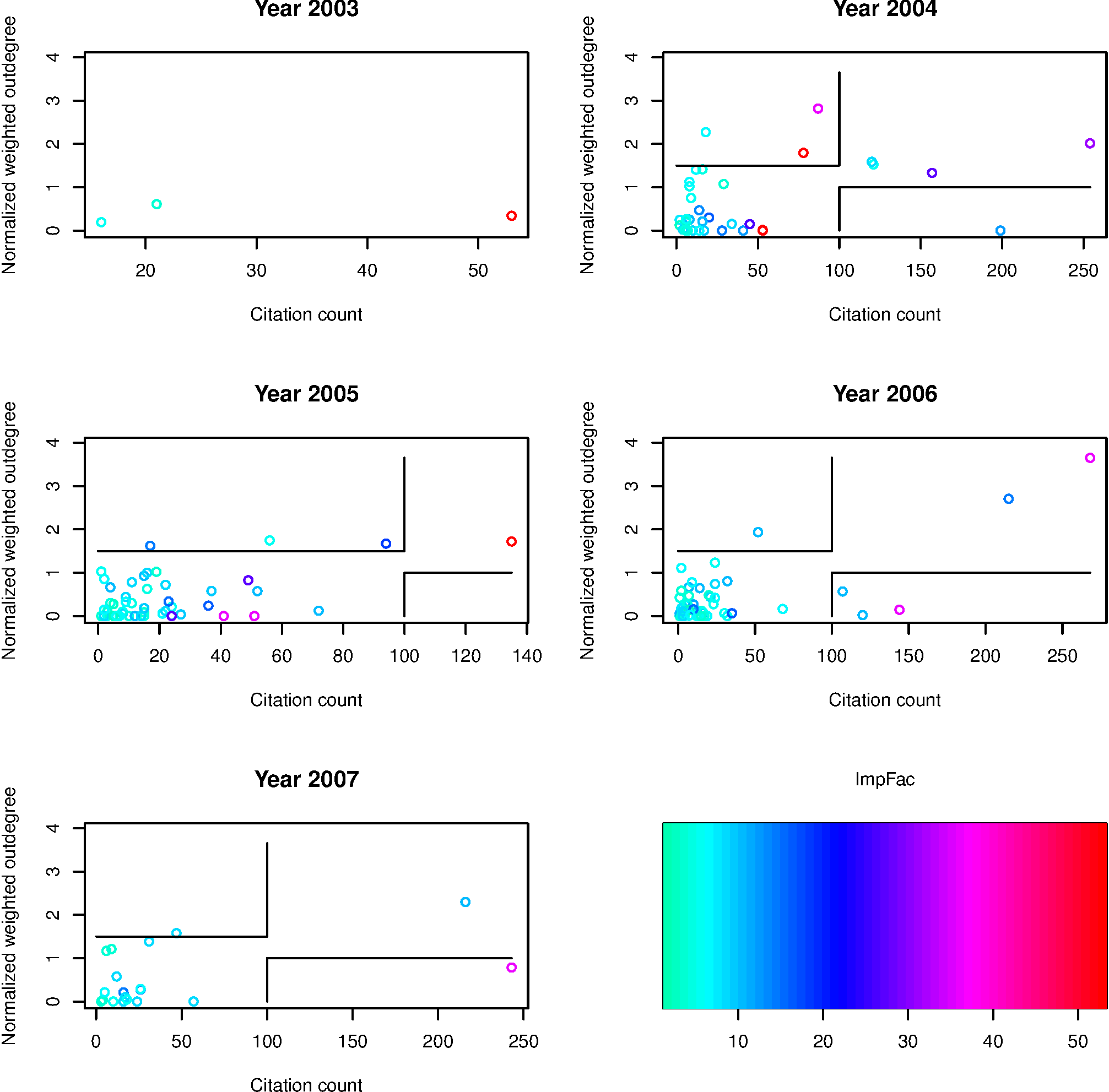

Supplement: Figure S1 — Stratified data-driven prediction of usefulness of datasets vs. their citation counts. Black solid lines mark the boundary for potentially interesting datasets; the boundaries are set to hold the same percentiles of data as in Fig. 3 in the main paper. ImpFac stands for Impact Factor of the publication venue. (TIFF) [file pone.0113053.s001.tiff]

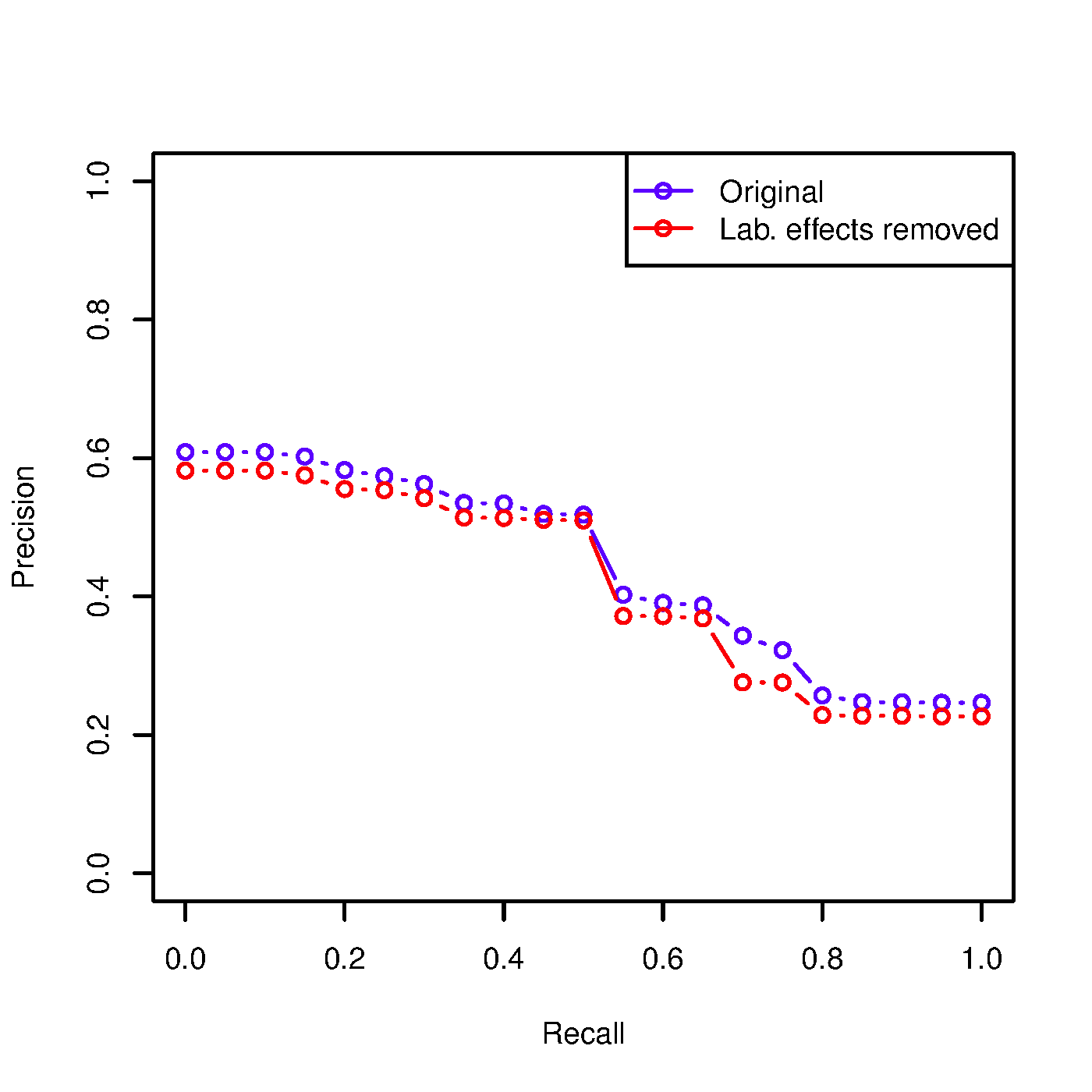

Supplement: Figure S2 — Removal of laboratory effects changes the retrieval performance only slightly, as measured by the precision-recall curves. Original: Replicated from Fig. 1 of the main paper; Lab. effects removed: all retrieval results from the same laboratory as the query data have been discarded. (TIFF) [file pone.0113053.s002.tiff]

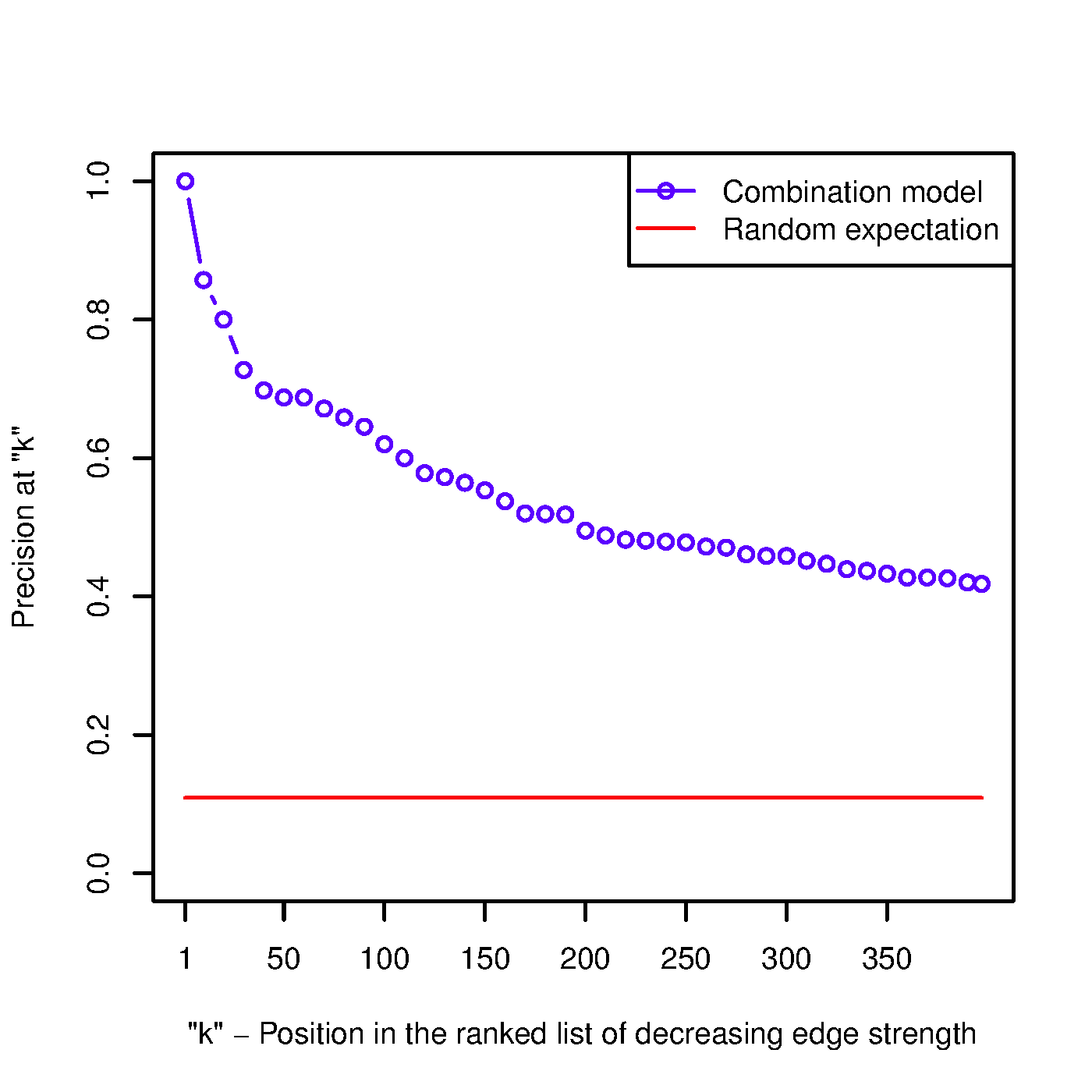

Supplement: Figure S3 — Overlap of data-driven recommendations with the actual citation graph: Precision for top edges that explain more than variation. The gold standard is the extended citation graph, which is built as the union of edges from 1) the original directed graph, 2) between any two articles that are cited together by some other article, and 3) between any two articles that have at least one common reference. (TIFF) [file pone.0113053.s003.tiff]

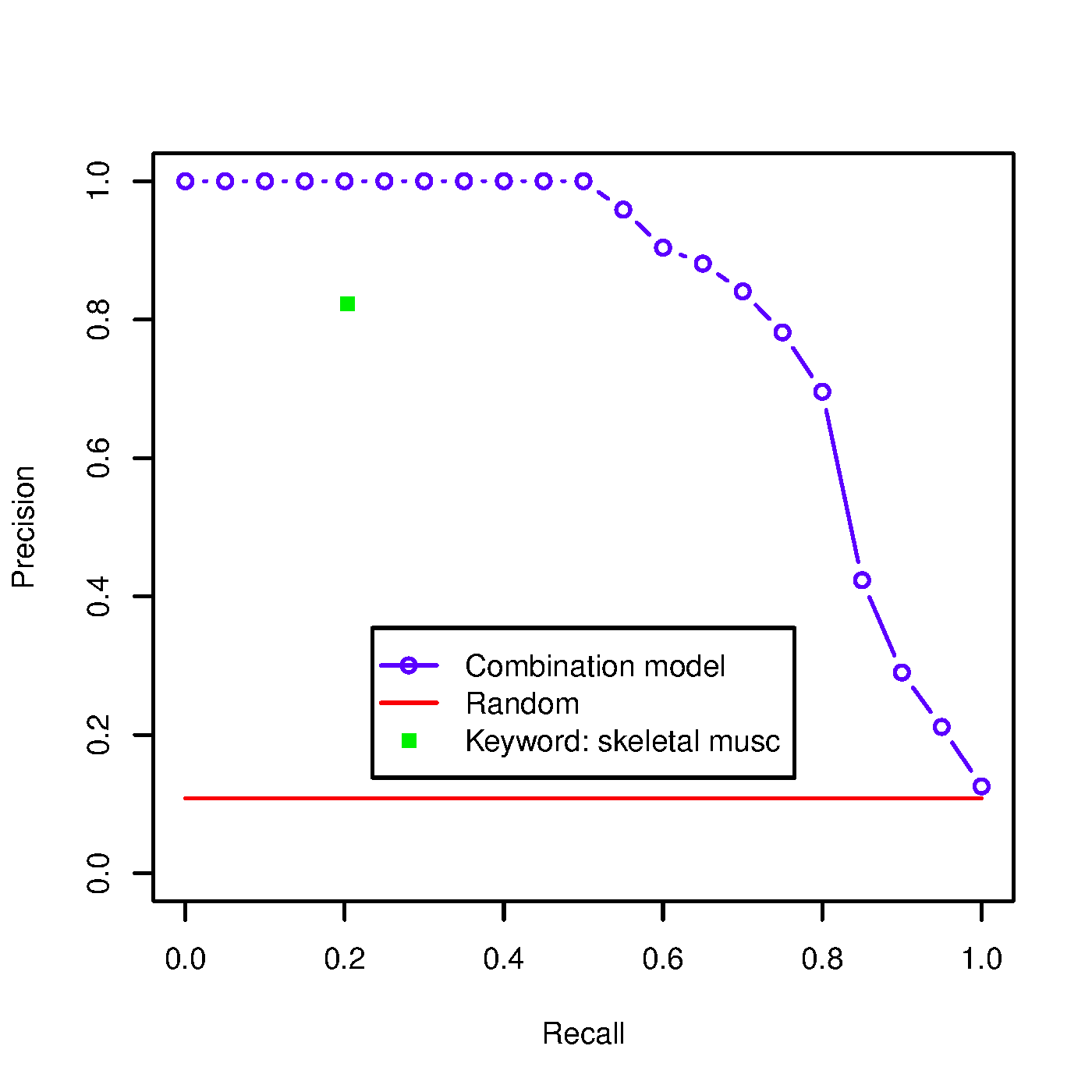

Supplement: Figure S4 — Retrieval performance evaluation of the data-driven model against keyword search in the skeletal muscle case study. The precision-recall curves are averaged across the 16 skeletal muscle datasets having at least 10 samples. (TIFF) [file pone.0113053.s004.tiff]
