# Supplementary material for: Toward Computational Cumulative Biology by Combining Models of Biological Datasets
Source: PLoS One. 2014 Nov 26;9(11):e113053. doi: 10.1371/journal.pone.0113053 (PMC4245117; doi:10.1371/journal.pone.0113053)
Supplement: Text S1 — More details on methods and results. (PDF) [file pone.0113053.s008.pdf]

Supporting Information Appendix: Toward computational cumulative biology by combining models of biological datasets

Ali Faisal *, **Jaakko Peltonen** * , **Elisabeth Georgii** * , **Johan Rung** † and **Samuel Kaski** * ‡

*Helsinki Institute for Information Technology HIIT, Department of Information and Computer Science, PO Box 15400, Aalto University, 00076 Aalto, Finland,†European Molecular Biology Laboratory, European Bioinformatics Institute (EMBL-EBI), Wellcome Trust Genome Campus Hinxton, CB10 1SD, UK, and ‡Helsinki Institute for Information Technology HIIT, Department of Computer Science, PO Box 68, 00014 University of Helsinki, Finland

Methods

Gene Set Enrichment Analysis. We used GSEA [1] to bring in biological knowledge in the form of pre-defined gene sets. GSEA starts by sorting genes with respect to their normalized expression levels. GSEA essentially consists of computing a running sum on the sorted list for each gene set; this running sum (enrichment score) increases when a gene belongs to the gene set and decreases otherwise; the final statistic is the maximum of this running sum. The procedure essentially amounts to computing a weighted Kolmogorov-Smirnov (KS) statistic. For each sample the KS statistic is normalized by dividing it by the mean of random KS statistics computed on randomly generated gene sets whose size is matched with the actual gene set; the 50 top-scoring gene sets are selected according to this normalized score. This simple thresholding ignores significance values but has been successfully used in earlier meta-analysis studies [2, 3, 4]; we earlier investigated the alternative of selecting gene sets based on a standard q-value cut-off (with $q < 0.05$), but it produced an excessively sparse encoding where more than 80% samples had no active gene sets [4]. The activity of each gene set is finally expressed as its core set or *leading edge subset*, consisting of genes found before the running KS score reached its maximum. We quantify the activity or a set simply by the size of the leading edge subset. It can be used analogously to the so called *bag-of-words* representations in text analysis, and we use it for the subsequent modeling of each dataset with the base models.

Base models. *Latent Dirichlet allocation* (LDA) [5, 6, 7] and *mixture of unigrams* [8] are probabilistic unsupervised models that give insight to datasets by describing them in terms of latent components. Each data sample, in our case quantified as gene set activities, is represented by a probability distribution over components (sometimes also called topics). The components are shared by all samples, but with different degrees of activation for each, and each component produces a characteristic distribution over the gene sets. In LDA each sample may be produced by multiple hidden components while the mixture of unigrams is a simplified version where each sample is assumed to come from a single component. The computational problem is to estimate the latent component structure (for each sample, the distribution over components and for each component, its distribution over gene sets) that has most likely generated the observed set of samples.

We use standard inference solutions for the models: collapsed Gibbs sampler for the LDA [7] and Expectation Maximization for the mixture of unigrams [8]. In LDA, the hyperparameters, the prior probability of each component, were optimized with Minka's stable fixed point iteration scheme [10], interleaved with the collapsed Gibbs sampling of the other parameters. The number of Gibbs iterations was 2500, found to yield good performance also earlier [6, 7, 9]. For the mixture of unigrams model, the maximum a posterior solution is estimated with the Expectation Maximization (EM) algorithm, using Laplace smoothing for the prior probabilities of the mixture components [8].

Model selection for base models. For each dataset we estimated the two base models and selected the one that best models the dataset. We split the dataset into two parts where 90% of the dataset samples were used for training the two models and the remaining 10% samples to compute the test-set predictive likelihood, a measure of how well the model fits the data. We repeated this procedure in a 10-fold cross-validation setup for each dataset and each of the two models. The model that performed better on average across the 10 folds was chosen to represent the dataset. Most datasets (74 out of 112) preferred the more expressive LDA model.

The number of components was selected with cross-validation for both models. We again used 10-fold cross-validation, separately for each dataset, to choose the number of components that lead to best predictive likelihoods on test samples from the same dataset. We observed that the optimal number of total topics could roughly be summarized by $\lceil N_D/3 \rceil$ for LDA and $\lfloor \sqrt{N_D} \rfloor$ for mixture of unigrams, where N_D is the total number of samples in a dataset. All predictive likelihoods (both for model selection and for retrieval) were computed using an empirical likelihood method (see [11]). For very small datasets ($N_D < 10$) within-set cross-validation is very noisy and we chose LDA which had been selected for the majority of datasets with $10 \le N_D \le 15$.

Strict Concavity of the objective function. For each query dataset q, there are multiple samples $i = 1, \ldots, N_q$. For each sample, each background dataset gives a probability. Let $\vec{x}_i = [p(x_i^q|M^{s_1}), p(x_i^q|M^{s_2}), \ldots, p(x_i^q|M^{s_{N_S}}), p(x_i^q|\Psi)]^\top$ be the column vector of probabilities for sample i from all background datasets and from the novelty model, where $^\top$ denotes vector transpose. Then the probability given by a mixture of background datasets is $\vec{\theta}^\top \vec{x}_i$ where $\vec{\theta}$ is the column vector of mixture weights (mixing proportions) over the background datasets and the novelty model. Since the mixing weights are mixture probabilities, they must lie in the canonical simplex denoted as

$$\Delta = \left\{ \theta | \theta_j \ge 0, \sum_{j=1}^{N_S+1} \theta_j = 1 \right\} . \quad [1]$$

Our optimization takes the form

$$\max_{\vec{\theta} \in \Delta} \log \left(\exp(-\lambda ||\vec{\theta}||^2) \prod_i \vec{\theta}^\top \vec{x}_i \right) = \max_{\vec{\theta} \in \Delta} \sum_i \log(\vec{\theta}^\top \vec{x}_i) - \lambda ||\vec{\theta}||^2 = \max_{\vec{\theta} \in \Delta} f(\vec{\theta}) \quad [2]$$

where the objective function is

$$f(\vec{\theta}) = \sum_i \log(\vec{\theta}^\top \vec{x}_i) - \lambda ||\vec{\theta}||^2 .$$

The objective $f(\vec{\theta})$ is a strictly concave function with respect to the multivariate parameter $\vec{\theta}$. This can be shown since the second

derivative of the function is negative. In detail, the function's gradient is

$$\nabla f(\vec{\theta}) = \sum_i \frac{\vec{x}_i}{\vec{\theta}^\top \vec{x}_i} - 2\lambda\vec{\theta} \qquad [3]$$

and the matrix of second-order partial derivatives (Hessian matrix) is

$$\nabla^2 f(\vec{\theta}) = -\sum_i \frac{\vec{x}_i\vec{x}_i^\top}{(\vec{\theta}^\top \vec{x}_i)^2} - 2\lambda I \qquad [4]$$

where I denotes the identity matrix. Since all elements of $\vec{x}_i$ are non-negative, it is easy to see the Hessian matrix is negative definite for all $\vec{\theta}$ where $\theta_i \geq 0$ as long as $\lambda > 0$. Therefore the objective function is strictly concave. A local maximum of a strictly concave function on a convex feasible region is the unique global maximum [12]; therefore maximizing the objective function to a local maximum by any algorithm yields the unique global maximum.

Maximizing the Objective Function. Maximization of concave functions on the unit simplex Δ can be done by the *Frank-Wolfe algorithm*. The algorithm performs the following steps.

Step 1: initialization. The algorithm initializes a solution as the vertex of the simplex having the largest objective value. A vertex of the simplex has $\theta_j = 1$ for some j and all other elements of $\vec{\theta}$ are zero. At vertex j, the objective function simplifies to

$$\sum_i \log(x_{ij}) - \lambda$$

where $x_{ij} = p(sample_i|dataset_j)$, which takes $O(N_q)$ time to evaluate per vertex. Thus creating the initialization takes linear $O(N_q N_S)$ time even with a simple brute-force evaluation of all vertices.

Step 2: Iteration. In each iteration, the algorithm improves the solution by two steps: (1) Find the maximal element j of the gradient. (2) Find the point along the line $\vec{\theta} + \alpha(\vec{e}(j) - \vec{\theta})$, $\alpha \in [0,1]$, which maximizes the objective function. Here $\vec{e}(j)$ means the vector where the element j is one and the others are zero. Computation of the gradient and finding the maximal element takes $O(N_q N_S)$ time.

Proof of Convergence and Scalability

Convergence Analysis. Since each iteration takes linear time with respect to the number of query samples and background datasets, the only remaining issue is the number of iterations required for good enough convergence. We now analyze the convergence properties of this iteration in two cases.

Case 1: optimal α. At first, we consider the case where the best value of α can be found along the line to a sufficient accuracy in a fixed amount of time, for example by restricting evaluations along the line to a fixed number.

Define a proportional regret function $h(\vec{\theta}) = (f(\vec{\theta}^*) - f(\vec{\theta}))/4C_f$ where $\vec{\theta}^*$ is the optimal parameter value maximizing the objective function, and $C_f \geq 0$ is a *measure of curvature* of f. In detail, C_f is defined as the largest quantity such that for all $\vec{\theta}_A \in \Delta$, $\vec{\theta}_B \in \Delta$, $\vec{\theta}_C$ where $\vec{\theta}_C = \vec{\theta}_A + \alpha(\vec{\theta}_B - \vec{\theta}_A)$ for some α, we have

$$f(\vec{\theta}_C) \geq f(\vec{\theta}_A) + (\vec{\theta}_C - \vec{\theta}_A)^\top \nabla f(\vec{\theta}_A) - \alpha^2 C_f \,.$$

With this notation, it can be shown ([13], Theorem 2.2) that at iteration k of the Frank-Wolfe algorithm the current solution $\vec{\theta}_k$ has regret

$$h(\vec{\theta}_k) \leq 1/(k+3)$$

and thus

$$f(\vec{\theta}^*) - f(\vec{\theta}_k) \leq 4C_f/(k+3) \,.$$

Thus, to achieve a desired regret ε, at most $4C_f/\varepsilon + 3$ iterations are needed independently of the number of background datasets; the amount of iterations needed depends only on the curvature.

Case 2: fixed α. It can even be shown that using a fixed value $\alpha_k = 2/(k+3)$ at each iteration k suffices to yield bounds for performance: with this choice of α_k the regret bound at iteration $k+1$ becomes ([13], Section 7)

$$h(\vec{\theta}_{k+1}) \leq 1/(k+4)$$

and thus

$$f(\vec{\theta}^*) - f(\vec{\theta}_{k+1}) \leq 4C_f/(k+4)$$

which again shows the number of iterations to achieve a desired regret does not depend on the number of background datasets, only on the curvature. It is enough to show the curvature is finite and does not depend on the number of background datasets; we now show this.

Analysis of the Curvature: As seen above, the smaller the curvature C_f, the better the bounds for the regret $f(\vec{\theta}^*) - f(\vec{\theta}_k)$ are. In our case the function f is twice differentiable and it can be shown ([13], Section 4.1) that

$$C_f \leq \sup_{\vec{\theta}_A \in \Delta, \vec{\theta}_B \in \Delta, \alpha \in [0,1]} -\frac{1}{2}(\vec{\theta}_B - \vec{\theta}_A)^\top \nabla^2 f(\vec{\theta}_\alpha)(\vec{\theta}_B - \vec{\theta}_A) \,.$$

where $\vec{\theta}_\alpha = \vec{\theta}_A + \alpha(\vec{\theta}_B - \vec{\theta}_A)$. For our cost function this becomes

$$\begin{aligned}
C_f \leq & \sup_{\vec{\theta}_A \in \Delta, \vec{\theta}_B \in \Delta, \alpha \in [0,1]} \frac{1}{2}(\vec{\theta}_B - \vec{\theta}_A)^\top \\
& \left(\sum_i \frac{\vec{x}_i\vec{x}_i^\top}{(\vec{\theta}_\alpha^\top \vec{x}_i)^2} + 2\lambda I\right)(\vec{\theta}_B - \vec{\theta}_A) \\
= & \sup_{\vec{\theta}_A \in \Delta, \vec{\theta}_B \in \Delta, \alpha \in [0,1]} \frac{1}{2} \\
& \left(\sum_i \frac{((\vec{\theta}_B - \vec{\theta}_A)^\top \vec{x}_i)^2}{(\vec{\theta}_\alpha^\top \vec{x}_i)^2} + 2\lambda ||\vec{\theta}_B - \vec{\theta}_A||^2\right) \\
\leq & \sup_{\vec{\theta}_A \in \Delta, \vec{\theta}_B \in \Delta, \alpha \in [0,1], 1 \leq i \leq N_q} \frac{1}{2}\left(N_q \frac{((\vec{\theta}_B - \vec{\theta}_A)^\top \vec{x}_i)^2}{(\vec{\theta}_\alpha^\top \vec{x}_i)^2} + 4\lambda\right) \\
\leq & \sup_{\vec{\theta}_A \in \Delta, \vec{\theta}_B \in \Delta, \alpha \in [0,1], 1 \leq i \leq N_q} \frac{1}{2}\left(N_q \frac{(\vec{\theta}_B \vec{x}_i)^2 + (\vec{\theta}_A^\top \vec{x}_i)^2}{(\vec{\theta}_\alpha^\top \vec{x}_i)^2} + 4\lambda\right) \\
\leq & \sup_{\vec{\theta}_A \in \Delta, \vec{\theta}_B \in \Delta, \alpha \in [0,1], 1 \leq i \leq N_q} \frac{1}{2}\left(N_q \frac{2(\max_j x_{ij})^2}{(\min_j x_{ij})^2} + 4\lambda\right) \qquad [5]
\end{aligned}$$

where for brevity we denoted $x_{ij} = p(x_i^q|M^{s_j})$ for $j = 1, \ldots, N_S$ and $x_{i,N_S+1} = p(x_i^q|\Psi)$. Notice that the right-hand side only depends on the maximal and minimal values that background datasets give to query samples, not on the number of such background datasets. Thus, as long as we ensure the maximal value is below some finite constant and the minimal value is above some small nonzero constant, the curvature C_f is finite and the convergence bounds of the Frank-Wolfe algorithm therefore do not depend on the number of background datasets. This condition is simple to ensure by suitable regularization of the models of background datasets.

Therefore under the simple condition that probabilities given to query samples are upper bounded and lower bounded above zero, the algorithm converges (towards the unique global maximum) to a desired tolerance of the regret in a finite number of iterations I, which can depend on the number of query samples but is independent of the size of number of background datasets.

Computational complexity. Our model needs to perform two main computation tasks for each new dataset: optimization of the objective function and computation of the predictive likelihoods. The optimization step needs to evaluate the function value, the gradient and maximal element of the gradient; the computation of the function value has complexity $O(N_q * N_S * O(\text{computing } p(x_i{}^q|M^{s_j}))$ while, as discussed in the previous section, computing the gradient and finding the maximal element takes linear $O(N_q * N_S)$ time in each iteration if a fixed step size is used (or if the number of line search evaluations is restricted below some fixed maximum). Thus the complete algorithm takes $O(I * N_q * N_S * O(\text{computing } p(x_i{}^q|M^{s_j})) + O(I * N_q * N_S)$. Clearly the dominating factor is the computation of the function value: $O(I * N_q * N_S * O(\text{computing } p(x_i{}^q|M^{s_j}))$.

The predictive likelihood for the query sample is computed as its average probability from $V = 1000$ multinomial distributions, estimated from randomly generated samples coming from the generative process of the earlier-trained model M^{s_j}; this is the standard *empirical likelihood scheme* discussed in [11]. The complexity of computing the predictive likelihood for the query dataset with G features (in our case gene-sets), given a model with T latent components is $O(G * V * T)$. Here T is upper bounded by the maximum number of samples in a background dataset. The total computational complexity is then simply the complexity for predictive likelihoods times the optimization scheme: $O(I * N_q * N_S * G * V * T)$. Since the number of iterations is independent of the number of background datasets, N_S, the complexity is linear with respect to it, $O(N_S)$, and therefore the model is reasonably tolerant to the fast growth of public repositories.

In our implementation a single query dataset took about 31 iterations and 0.15 seconds on average on an Intel® Core (TM) i7 CPU @ 2.93GHz, to find the optimal weight vector.

Results

Normalization of citation counts. Older datasets tend to have higher citation counts and outdegrees; in Fig. 3 we removed this bias by a normalization technique, and identified interesting datasets having very low citation counts and very high outdegrees or vice versa. To verify the analysis results (identified datasets) are not artifacts of the normalization, we reanalyzed the original data without applying citation count normalization. Fig. S1 shows the result as stratified subfigures plotted for each year separately. The datasets identified using the original citation counts (top-left and bottom-right corners in each subfigure of Fig. S1) are an exact match with the datasets identified after normalization.

Retrieval performance after discounting for the laboratory effect. In microarray experiments laboratory effects are known to be strong [14]. The 206 datasets studied were generated from 163 laboratories. The top laboratory was responsible for 7 datasets, whereas 142 laboraties only contributed a single set. To test how much the laboratory effects have affected our results, we discarded all retrieved results from the same laboratory as the query set. The original precision-recall curve and the corrected curve are in Fig. S2; the mean average precision dropped to 0.44 from 0.42. The small change in performance shows that our result is mainly due to other effects captured by the model than the laboratory effect.

Quantitative comparison of data-driven results against the citation patterns. Of the 23 direct citation links, eleven were also found by our model as having a non-zero edge weight. Six links could not have been found; five of them are citations by papers having very small datasets (< 10), which we had considered to be too small to act as queries while one citation link is between datasets released on the same date. Of the remaining six links not observed in our model, two are the cross-cluster citations that are not due to biological similarity of the datasets as discussed in the paper; two cell line datasets about multiple myeloma and large cell lymphoma (GSE6205 and GSE6184 respectively) cite a leukemia study (GSE2113) where plasma cells are profiled; one dataset measuring HIV infection from T cells (GSE6740) cites a very small dataset about thymocyte, the limited size of the set reducing its corresponding model's relative capability to explain other sets compared to large datasets in the collection. Finally, E-TABM-26, a prostate cancer study, cites E-MEXP-156 (a study about tumorigenic and nontumorigenic Human Embryonic Kidney Cells) in the context of cell apoptosis of cancer cells in general.

Vice versa, we evaluated the top-weight data-driven edges against direct and indirect citation links and found a favorable, non-random overlap. For this we use a modern standard metric in information retrieval called precision @k which measures precision at each position k in the ranked list of top retrieval results from a search engine. In our case the results are the ranked list of inferred edges in descending order of their edge strengths. The citation patterns were used as the gold standard for existence of an edge between two datasets using their corresponding publication information. Fig. S3 shows that the precision @k is reasonably high; for the top two hundred data-driven edges (*i.e.*, $k = 200$) the value is 0.5.

Densely connected set of experiments in the relevance network. For each of the top 20 strongest edges in the relevance network (listed in Table S1) we searched for associated cliques, *i.e.*, connections within neighbors of the edge, where the clique size is at least three. We found seven cliques; four of them (breast cancer, leukocytes, human immune T helper cells and developmental stages of thymocytes) are described in the main text. The remaining three are an adenocarcinoma, a brain tissue and a skeletal muscle clique. The first clique is among GSE4824, GSE5258 and GSE6914; all three datasets are heterogeneous collections of different cancerous tissues where majority of the samples are cell line profiles from either lung or breast adenocarcinoma. The second clique is among GSE1297, GSE5392 and E-MEXP-114 that profile normal and diseased brain tissue. The last clique is among all skeletal muscle experiments of the collection where the strongest edge is between GSE3307 and GSE6011 that both measure Duchenne muscular dystrophy sampled from quadriceps muscle tissues, the former also containing samples form other skeletal muscle diseases.

Skeletal muscle retrieval case study. We lastly present a case study to illustrate how the model retrieval can support a researcher in finding relevant data on a specific topic, lessening the need for laborious manual searches. The topic of interest was gene expression in skeletal muscle and our database consisted of the human gene expression atlas [15], which includes eight skeletal muscle datasets among a total of 206 datasets, plus additional 16 skeletal muscle datasets extracted manually from ArrayExpress (Table S2). First, we tested how well the data-driven method retrieved other skeletal muscle datasets when querying with any single skeletal muscle dataset. The retrieval performance across all 16 query datasets was close to optimal, whereas keyword searches were not as good in the same task (Fig. S4). The reason for that was the lack of a consistent annotation vocabulary in the dataset descriptions; in particular, different levels of specificity had been used.

Next we looked in more detail into the retrieval results of the individual queries. For all of them, the retrieval result was sparse, *i.e.*, less than 10% of the datasets were found to be relevant to the query (by a non-zero weight). We further investigated the ranking of results provided by the data-driven retrieval model. For 12 queries all retrieved results were other skeletal muscle datasets; results for the remaining queries contained at least one false positive, and they are summarized in Table S3. The top retrieved non-skeletal muscle dataset is the brain tissue dataset GSE5392, followed by the kidney dataset GSE781. Kidney does not have a direct biological connection to skeletal muscle. It is known that some areas of kidney are rich in

blood vessels, which are lined by smooth muscle. Skeletal muscle, smooth muscle and cardiac muscle are the three main muscle types in the human body. Interestingly, one skeletal muscle datasets, E-MEXP-216, was never retrieved by any skeletal muscle query, which suggests that it is an outlier in some respect. The dataset contains a combination of human and macacque liver and skeletal muscle samples. It contains only four human skeletal muscle samples.

Finally, the internal ranking of skeletal muscle datasets in response to a particular query dataset seems to be guided to a large extent by health conditions:

- **E-GEOD-9397** contains samples annotated with disease status FSHD (Facioscapulohumeral muscular dystrophy). The top retrieved dataset for that query is E-GEOD-10760, the only other dataset in the collection annotated with FSHD.
- The top retrieved result for **E-GEOD-12648** is **E-GEOD-11686** and vice versa. Both of them are neuromuscular disorders, hereditary inclusion body myopathy (HIBM) and cerebral palsy, and they are the only datasets with these specific diseases in our data collection.
- **E-GEOD-1786** partially contains samples from COPD (Chronic Obstructive Pulmonary Disease) subjects; the disease has a muscle wasting effect. The top retrieved result is E-GEOD-10760; it contains samples from the same muscle type, but another disease (FSHD) which also leads to muscle wasting.
- **E-GEOD-1295** contains samples of the trained and untrained muscle of non-young overweight people with prediabetic metabolic syndrome. Half of the samples in E-GEOD-1786 are also from trained muscles (of COPD patients and controls, old overweight men). E-GEOD-1786 appears at rank 6, and it is the only background dataset known to contain trained muscle samples. Among the top 5 datasets, 3 are annotated to contain disease samples related to weakening of muscles, another one is known to contain old overweight samples (**E-GEOD-8441**).

In summary, disease conditions and health states of tissue seem to determine the ranking within datasets of the same tissue (sub)type.

1. Subramanian A, et al. (2005) Gene set enrichment analysis: A knowledge-based approach for interpreting genome-wide expression profiles. *Proc Natl Acad Sci U S A* 102:15545–15550.
2. Segal E, et al. (2004) A module map showing conditional activity of expression modules in cancer. *Nat Genet* 36:1090–1098.
3. Caldas J, Gehlenborg N, Faisal A, Brazma A, Kaski S (2009) Probabilistic retrieval and visualization of biologically relevant microarray experiments. *Bioinformatics* 25:i145–i153.
4. Caldas J, et al. (2012) Data-driven information retrieval in heterogeneous collections of transcriptomics data links *SIM2s* to malignant pleural mesothelioma. *Bioinformatics* 28:i246–i253.
5. Pritchard J-K, Stephens M, Donnelly P (2000) Inference of population structure using multilocus genotype data. *Genetics* 155:945–959.
6. Blei D-M, Ng A-Y, Jordan M-I, Lafferty J (2003) Latent Dirichlet allocation. *J Mach Learn Res* 3:993–1022.
7. Griffiths T-L, Steyvers M (2004) Finding scientific topics. *Proc Natl Acad Sci U S A* 101:5228–5235.
8. Nigam K, Mccallum A-K, Thrun S, Mitchell T (2000) Text classification from labeled and unlabeled documents using EM. *Machine Learning* 39:103–134.
9. Teh Y-W, Jordan M-I, Beal M-J, Blei D-M (2006) Hierarchical Dirichlet Processes. *J Am Stat Assoc* 101:1566–1581.
10. Minka T-P (2000) Estimating a Dirichlet distribution. *Technical report* available at http://research.microsoft.com/en-us/um/people/minka/papers/dirichlet/.
11. Li W, McCallum A (2006) Pachinko allocation: DAG-structured mixture models of topic correlations. *Proceedings of the Twenty-Third international conference on Machine learning* (ACM New York, USA) pp 577–584.
12. Bradley S, Hax A, Magnanti T (1977) *Applied Mathematical Programming*. Addison-Wesley.
13. Clarkson, K L (2010) Coresets, sparse greedy approximation, and the Frank-Wolfe algorithm. *ACM Transactions on Algorithms* 6(4): Article 63.
14. Zilliox M-J, Irizarry R-A (2007) A gene expression bar code for microarray data. *Nat Methods* 4:911–913.
15. Lukk M, et al. (2010) A global map of human gene expression. *Nat Biotechnol* 28:322–324.
16. Shannon P, et al. (2003) Cytoscape: a software environment for integrated models of biomolecular interaction networks. *Genome Res* 13:2498-2504.

Figure and Table Legends

Fig. S1: Stratified data-driven prediction of usefulness of datasets vs. their citation counts. Black solid lines mark the boundary for potentially interesting datasets; the boundaries are set to hold the same percentiles of data as in Fig. 3 in the main paper. *ImpFac* stands for Impact Factor of the publication venue.

Fig. S2: Removal of laboratory effects changes the retrieval performance only slightly, as measured by the precision-recall curves. *Original:* Replicated from Fig. 1 of the main paper; *Lab. effects removed:* all retrieval results from the same laboratory as the query data have been discarded.

Fig. S3: Overlap of data-driven recommendations with the actual citation graph: Precision @k for top edges that explain more than 2.5% variation. The gold standard is the extended citation graph which is built as the union of edges from 1) the original directed graph, 2) between any two articles that are cited together by some other article and 3) between any two articles that have at least one common reference.

Fig. S4: Retrieval performance evaluation of the data-driven model against keyword search in the skeletal muscle case study. The precision-recall curves are averaged across the 16 skeletal muscle datasets having at least 10 samples.

Table S1: Top 20 strongest edges in the relevance network.

Table S2: ArrayExpress accession numbers of 16 skeletal muscle datasets used in the retrieval case study in addition to the human gene expression atlas [15]. All datasets were measured with the human genome platform HG-U133A, the same used in the atlas.

Table S3: Skeletal muscle queries with at least one retrieved non-skeletal muscle dataset, sorted according to decreasing precision.
